# Relating transcriptomics to protein abundance reveals self‐driven versus interactor‐driven proteins

**DOI:** 10.1002/pro.70806

**Published:** 2026-09-16

**Authors:** Loulwah Arnaout, Volkhard Helms, Aram Papazian

**Affiliations:** ^1^ Center for Bioinformatics Saarland Informatics Campus, Saarland University Saarbruecken Germany

**Keywords:** autoencoder, linear regression, protein complex, protein‐protein interactions, proteomics, RNA half‐life, transcriptomics

## Abstract

Statistically modeling the interdependence between transcriptomics and protein abundances remains a persistent challenge in bioinformatics research. Transcriptomic data and proteomic abundances typically display a moderate Pearson's correlation of about 0.5, while in tumor conditions, the correlation may decrease to a much weaker correlation of 0.2. Given that transcriptomic datasets are commonly analyzed due to their ample availability, it is imperative to understand this correlation in greater detail and to be aware of potential deficiencies while conducting research connecting transcriptomic data to protein abundances. Recently, the Clinical Proteome Tumor Analysis Consortium has collected several large‐scale proteomic datasets containing transcriptomic and proteomic abundances from hundreds of patients in The Cancer Genome Analysis cohorts. Utilizing these data, a detailed analysis of how protein and transcript abundances correspond for individual genes was presented. As variables of linear regression models, transcript levels of each gene and of their known protein–protein interaction partners, taken from the STRING database, are used. Interestingly, this resulted in two distinct classifications of genes, with a continuum present in between: “self‐driven” genes, for which protein abundances are mainly determined by the gene's own transcript level, as well as “interaction‐driven” genes, for which transcript levels of other genes possess far greater relevance for its protein abundance than its own transcript level. Notably, the former displays significantly shorter mRNA half‐lives than the latter. Namely, this is observed in the ribosome, which is encoded by rather long‐lived mRNAs and displays protein abundance levels that are determined by the transcript levels of the other protein components, rather than by its own transcript level. These findings suggest that biomarker interpretation and therapeutic intervention should consider whether a gene is self‐driven or interactor‐driven, as protein abundance in the latter may be regulated primarily through interaction partners rather than the gene's own transcript level.

## 1 INTRODUCTION

Proteins are the primary effectors of cellular function: defining how their abundances are regulated is paramount to differentiating hallmarks of healthy tissue and malignancy. Although cancer is initiated by genomic alterations, the resulting changes in protein abundance play a major role in driving key biological processes underlying tumor growth and progression, shaping patient survival (Ősz et al., 2021) and response to therapy (Yu et al., 2016).

Despite the central role of analyzing proteomics in cancer biology, quantifying protein abundances at larger scale remains technically demanding and costly, rendering transcriptomic measurements a common proxy for proteomic states. However, transcript levels do not reflect protein abundances fully, given that there is only a moderate correlation present (Liu et al., 2016; Maier et al., 2009). Across large‐scale datasets, the squared Pearson correlation coefficient between mRNA transcripts and protein levels centers at approximately 0.4, indicating that transcript levels explain roughly 40% of the variation in protein abundance (Vogel & Marcotte, 2012).

The remaining variation reflects both technical and biological factors. Certainly, experimental reproducibility and measurement noise introduce substantial variance, limiting the observed mRNA‐protein correlation in tumor proteomic profiles (Upadhya & Ryan, 2022), yet the biological component of this discordance arises from regulatory processes that operate beyond transcription itself. Protein abundance is shaped by multiple layers of post‐transcriptional, translational, and post‐translational control, including mRNA stability, translation efficiency, protein folding, complex assembly, post‐translational modifications, and targeted degradation. These processes can significantly contribute to determining final protein concentrations as much as transcriptional regulation (Vogel & Marcotte, 2012).

These regulatory layers suggest that information relevant to protein abundance may not reside solely at the transcript level. The function of proteins is highly interdependent within complexes and pathways, such that the abundance or stability of one component can depend on the state of others. Thus, transcripts of known interacting proteins may carry cues about the regulatory context influencing a protein's abundance. Databases such as STRING (Szklarczyk et al., 2019) compile experimentally supported and computationally inferred interaction partners that could be used to contextualize each protein's regulatory environment.

Several recent studies have evaluated the extent to which protein abundance can be predicted from transcriptomic data. A community benchmarking study (Yang et al., 2020) was conducted utilizing transcript levels and gene‐copy numbers of ovarian and breast cancer datasets, comparing multiple machine learning approaches to find that transcript levels alone achieve moderate accuracy. Srivastava et al. (2022) further investigated the role of incorporating transcripts of known interactors to improve prediction accuracy, which proved favorable. Using matched mRNA‐protein profiles across eight CPTAC cancer types (Edwards et al., 2015), the authors compared models trained on a gene's own transcript to models that additionally incorporated transcripts of protein interaction partners that were procured from Comprehensive Resource of Mammalian Protein Complexes (CORUM) Database (Giurgiu et al., 2019) complexes or STRING (Szklarczyk et al., 2019) interaction networks. Srivastava and colleagues found that including interactor transcripts yielded improved prediction performance for many proteins, highlighting that trans features can capture regulatory influences that are not gleaned from the gene's own transcript alone.

In this study, we sought to assess not only whether transcripts of additional genes, such as interaction partners, could provide incremental predictive value, but also to investigate whether gene‐specific regression models could serve as a framework for interpreting how different transcriptomic features correlate to protein abundances. Using matched mRNA‐protein data from 10 CPTAC cancer cohorts, we trained a separate linear model for each gene, allowing its protein abundance to be predicted from the expression levels of its own transcripts as well as the transcripts of other genes. By comparing models built with annotated interaction partners to models built with randomly selected genes, the degree of biological structure these features capture beyond what is expected from arbitrary transcript combinations is evaluated.

This gene‐level modeling approach additionally provides a unique opportunity to examine the coefficients themselves: as each model yields a set of estimated influences linking transcripts to proteins, the full collection of coefficients forms a proteome‐wide map entailing how transcriptomic variation relates to proteomic outcomes. These coefficient patterns were analyzed to identify signatures consistent with protein complexes, cancer hallmark genes, and KEGG (Kanehisa et al., 2019) pathways; latent embeddings were implemented to explore higher‐level structure in how transcripts shape protein abundance across the proteome.

## 2 RESULTS

### 2.1 Transcriptomic features predict protein abundance

**FIGURE 1:**
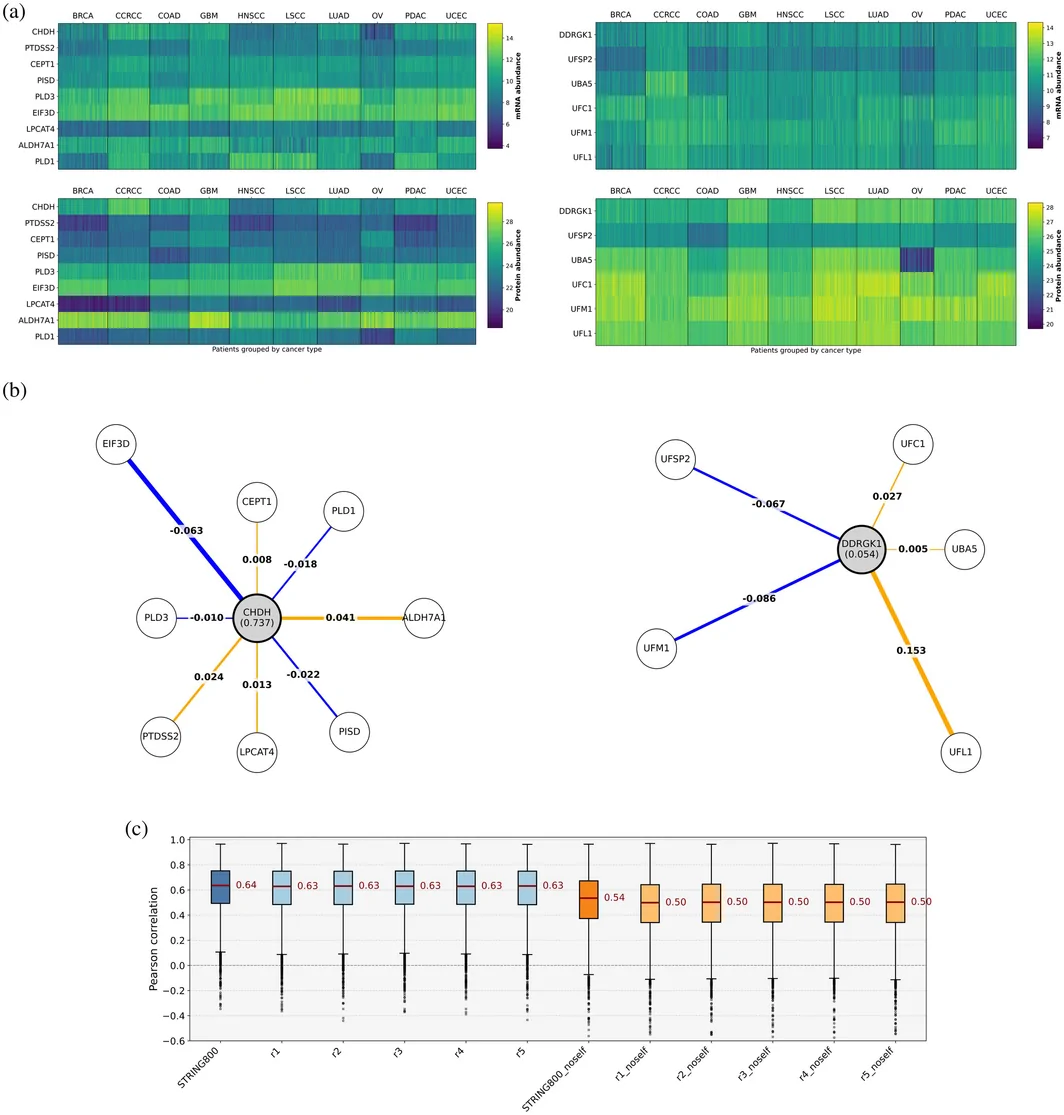
Linear regression models predict proteomic abundance from interaction‐aware transcriptomics. (a) mRNA (top) and protein (bottom) abundances of two selected genes, CHDH (left) and DDRGK1 (right) together with their STRING interactors across tumor samples, grouped by cancer type. (b) Linear models derived for CHDH (self‐driven) and DDRGK1 (interactor‐driven). The center node shows each gene's self‐coefficient; edges show interactor coefficients, with color indicating direction (blue = negative, orange = positive), as well as the width and distance from the center node reflecting magnitude. (c) Performance of all gene‐models (*N* = 5447 per box) using STRING interactors vs. random genes as predictors. Blue boxes include the gene's own transcript, while the orange boxes exclude the gene's own transcripts. Lighter‐colored boxes correspond to random‐feature controls. Median Pearson correlations are listed in red to the right of each box.

Data from 10 various cancer types were extracted from the CPTAC and filtered to include only patients with matched transcriptomic and proteomic profiles, resulting in a total of 1351 samples. Transcriptomic and proteomic abundances of each gene across these samples showed a mean Pearson's correlation of 0.36. To provide a qualitative view of the input data and expression patterns across the analyzed samples, the transcript and protein abundances of two example genes, along with their corresponding STRING‐annotated interactors, were visualized in Figure 1a. In the figure, each heatmap pair displays mRNA abundances, shown in the top row, and protein abundances, shown in the bottom row, across all 1351 samples, ordered by cancer type. The left panel corresponds to the gene product of CHDH and its 7 STRING interaction partners, while the right panel depicts DDRGK1 and its 6 interactors. In the CHDH heatmaps to the left of Figure 1a, several interactors display relatively stable transcript and protein abundances across most cancer types. Namely, PLD3 and EIF3D show consistent patterns in high transcript and protein abundances across samples. In the DDRGK1 heatmaps to the right, genes display more tumor‐specific expression patterns, such as low protein abundance levels of UBA5 in ovarian cancer samples.

As further detailed in the Methods, this datatype was utilized for a shared set of 5447 genes to evaluate whether incorporating the transcript levels of other genes, independent of a gene's own mRNA, enables more accurate prediction of protein abundances across various cancer types. For each gene, a separate linear regression model was trained, using the gene's own transcript level in conjunction with the transcript levels of its STRING‐annotated interactors, or for comparison, randomly selected genes from our analysis set, equivalent to the number of interactors, as predictors for the model. In addition, the regression models also accounted for two categorical features: cancer_type (indicating the cohort from which each sample originated) and disease_status (specifying whether the sample was derived from healthy or tumor tissue). These features account for cohort‐specific technical variation and biological differences between healthy and tumor samples. A more detailed outline of the model architecture and predictors is described in the Methods.

In Figure 1b, interactor‐based model representations of the two example genes of Figure 1a are illustrated to demonstrate how genes can rely on their own transcripts vis‐a‐vis those of their interactors. CHDH represents a “self‐driven” gene, whose own transcript provides most of the predictive signal: its self‐coefficient score of 0.737 is more than two‐fold the combined coefficients of its interactors. In contrast, DDRGK1 represents an “interactor‐driven” gene, where most of its protein variation is attributed to partner transcripts, with the self‐transcript playing a less significant role. In line with this distinction, CHDH shows consistently strong transcript‐protein correlations across cancer types, with an average Pearson correlation of 0.86, while the correlation is only 0.16 for DDRGK1, reinforcing their classifications as self‐driven and interactor‐driven genes, respectively.Furthermore, a complete representation was generated, integrating the 4769 gene‐level models, a subset of the 5447 gene set in which each model possesses at least 1 interactor, into a single directed network. In this network, each gene corresponds to a node and edges represent the learned coefficients between genes: a directed edge from gene X to gene Y encodes the estimated influence of X's transcript abundance on the protein abundance of Y, measured by the coefficient of feature X in gene Y's model. Edge weights capture the magnitude of this effect, while edge colors indicate its direction, either as positive or negative. To support interpretability, each edge is further annotated with the *p*‐value and 95% confidence interval of the estimated coefficient and a variance inflation factor (VIF), providing diagnostics for coefficient significance and multicollinearity; see Methods, Section 4.2, as well as Supplementary Figure S1. Nodes are colored according to the model's performance, as indicated by the Pearson correlation, allowing for simultaneous visualization of predictive strength and network connectivity. Nodes are also annotated with the number of samples for which the gene's transcript abundance is exactly zero. Due to standardization mapping zero values to negative values, frequent zeros could blur coefficient interpretation. However, most genes that were considered exhibited few or no zero values, making this effect negligible for the majority of models. This directed network offers a complementary view of proteomic regulation such that unlike traditional protein–protein interaction networks with binary “interacts/does not interact” relationships, this network encodes the estimated direction and strength of transcript‐level influences on protein abundances. The full network can be found in the Supplementary Information.

Across 1351 samples, the interactor‐based models achieved a median cross‐validated Pearson correlation of 0.64 between observed and predicted protein abundances, with the performance distribution illustrated by the leftmost dark blue box plot in Figure 1c. To assess the contribution of the gene's own transcript, additional models were trained that used only the transcripts of STRING‐annotated interactors, omitting the self‐transcript. These models output a considerable performance drop, reaching a median cross‐validated Pearson correlation of 0.54, pictured by the dark orange box plot in Figure 1c.

To determine whether interaction‐based features provide specific predictive value, the performance of interactor‐feature models was compared to random‐feature models, in which interactors were replaced by an equal number of randomly selected genes from the set.

When the self‐transcripts were included, the difference between STRING‐based and random models was minimal, with a change in median Pearson correlation from 0.64 to 0.63; see Figure 1c. In accordance with this finding, for 79% of genes with at least one interactor, the self‐transcript was the strongest predictor, with its coefficient being, on average, approximately 3.4 times larger than the strongest interactor. When the self‐transcript was excluded, the gap between STRING‐based and random models became slightly more apparent, with the median Pearson correlation dropping from 0.54 to 0.50, indicating that transcripts of annotated interaction partners may carry somewhat more relevant information than arbitrary transcripts. To further assess this difference, 100 random‐feature models were additionally generated for each gene. Comparing the STRING‐based model performance for each gene with the mean performance across its 100 random models revealed a significant overall shift toward higher performance for the STRING‐based models (one‐sided Wilcoxon signed‐rank test, $p=7.45\times 10^{-157}$). For visualization, Figure 1c shows five representative random runs. Though offering a modest improvement, excluding self‐transcripts supports the choice to use STRING‐derived interactors for downstream analyses, which offers a biologically grounded and interpretable predictor set that allows the resulting coefficients to reflect meaningful relationships, as opposed to arbitrary transcript associations.

In addition to linear regression, a nonlinear modeling approach was evaluated using a Random Forest regressor, which was trained on the same set of variables as the previous model. The interactor‐based and random‐feature models were trained both with and without inclusion of the self‐transcript. This distinction between interactor‐based and random models was ultimately comparable to the results observed with linear regression. When the self‐transcript was included, Random Forest models achieved a median Pearson correlation of 0.58, as opposed to 0.57 for random‐feature models. On the other hand, when the self‐transcript was excluded, the difference became slightly more pronounced, with median Pearson correlations of 0.49 and 0.46 for the interactor‐based and random‐feature models, respectively; see Supplementary Figure S2.

### 2.2 Self‐ and interactor‐driven genes exhibit distinct regulatory properties

Extending this analysis, transcript‐protein correlations were compared across all self‐driven and interactor‐driven genes, as classified according to Methods, Section 4.3.4. Self‐driven genes with larger self‐coefficients showed higher transcript‐to‐protein correlations, with a median correlation of 0.58, while interactor‐driven genes exhibited lower correlations with a median of 0.17. Via a one‐sided Mann–Whitney U‐test, it was determined that the difference was highly significant ($p=2.65\times 10^{-100}$), indicating that interactor‐driven genes tend to display weaker coupling between transcript and protein abundance.

To further characterize these two groups, we also analyzed the classification of their interaction partners. Note that self‐driven and interactor‐driven genes represent two extreme classes defined by our coefficient thresholds, while the remaining 3075 of the 4025 eligible genes (those with at least three interaction partners) did not satisfy either criterion and were therefore classified as neither. Interaction‐driven genes were connected predominantly to other interaction‐driven genes: 47.1% of their interaction partners were themselves classified as interactor‐driven, whereas only 0.5% were self‐driven, with the remaining 52.5% classified as neither. In contrast, self‐driven genes showed much smaller fractions of both self‐driven (7.1%) and interactor‐driven (7.6%) interaction partners. Accordingly, interaction‐driven genes contained a significantly higher fraction of interaction‐driven partners than other genes (Mann–Whitney U test, $p=3\times 10^{-122}$).

To assess the robustness of the coefficients underlying these classifications, bootstrap 95% confidence intervals were computed for all regression coefficients. For the defining coefficient of every classified gene, namely the self‐coefficient for self‐driven genes and the strongest non‐self coefficient for interactor‐driven genes, the confidence interval remained entirely above or below zero. Thus, all 410 self‐driven genes and all 540 interactor‐driven genes were classified based on sign‐stable coefficients.

RNA stability was subsequently compared between the self‐driven and interactor‐driven groups using the RNA half‐life measurements described in Methods Section 4.3.6 (Vock et al., 2025). After restricting the analysis to genes with available RNA half‐life measurements, 398 self‐driven and 504 interactor‐driven genes remained. Interactor‐driven genes had substantially longer‐lived transcripts, with a median RNA half‐life of 6.47 h, compared with 3.87 h for self‐driven genes. This difference was highly significant according to a one‐sided Mann–Whitney U test ($p=2.56\times 10^{-18}$; Supplementary Figure S8a).

### 2.3 Protein complex coordination

After establishing overall model performance, we investigated whether the learned coefficients reflected known biological structure by asking whether members of known protein complexes act in a coordinated manner within our transcript‐protein models. Using CORUM annotations, the focus was shifted to protein complexes possessing at least three subunits present in the dataset; then, both their internal interactions and influences on genes outside of the complex were evaluated.

**FIGURE 2:**
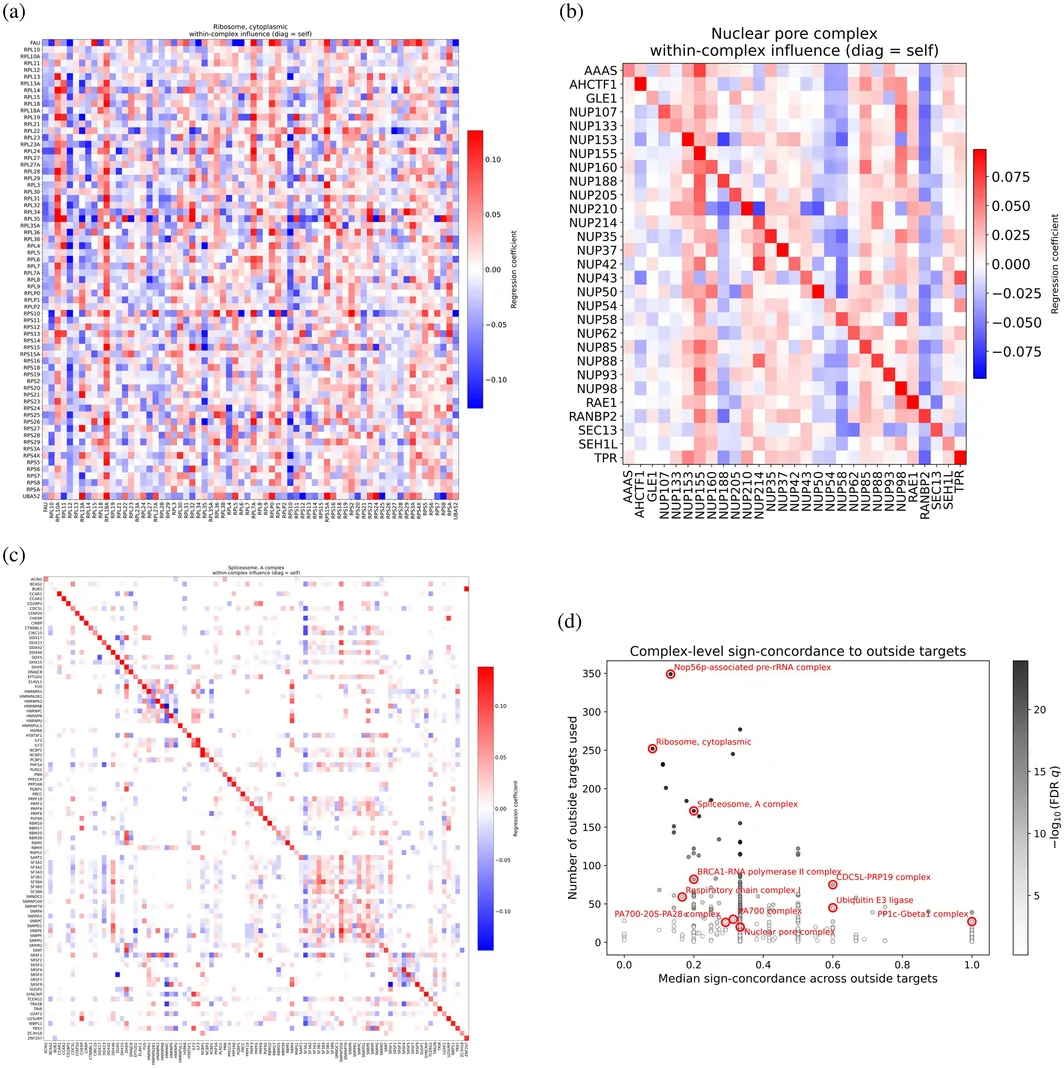
Protein complexes exhibit coordinated regulatory influence on external targets. (a)–(c), Within‐complex coefficient matrices for the cytoplasmic ribosome, nuclear pore complex, and spliceosome A complex, respectively. Rows are gene‐specific models and columns are transcript features. The colors denote whether the coefficient values are positive (in red) or negative (in blue). The diagonal entries show self‐coefficients. (d) Median sign‐concordance scores across outside targets for all 956 complexes, plotted against the number of outside targets with non‐zero coefficients. Point color, ranging from white to black, encodes significance from a one‐sided Wilcoxon test.

First, eight complexes were selected from the top 2% of the most represented complexes in the dataset: the Nop56p‐associated pre‐rRNA complex, the cytoplasmic ribosome, spliceosome A complex, BRCA1‐RNA Pol II complex, respiratory chain complex I, PA700 complex, PA700‐20S‐PA28 complex, and the nuclear pore complex. For illustrative purposes, the full matrix of within‐complex coefficients is visualized for three representative complexes—the nuclear pore complex, cytoplasmic ribosome, and spliceosome A complex—in Figure 2a–c. The subsequent analyses then addressed three research questions concerning protein complex coordination across the selected complexes, as listed in the subsection titles.

#### 2.3.1 Do transcripts of complex members tend to positively influence each other's protein levels?

Only two of the eight complexes featured demonstrated statistically significant positive within‐complex effects: the nuclear pore complex (adjusted $p=9.7\times 10^{-5}$) and the Spliceosome A complex (adjusted p=0.0294). Despite these significant results, heatmaps in Figure 2b,c include both negative and positive edges, likely attributed to the heatmap colors reflecting individual regression coefficients, while statistical tests summarize the overall direction and magnitude across members. Therefore, coordinated behavior does not require uniformly positive heatmap entries.

To evaluate whether coordinated behavior could be masked by averaging across functionally distinct complex members, the analysis was repeated using only inferred core‐like proteins, identified by transcript co‐expression as described in Methods Section 4.3.1. Under this restriction, the cytoplasmic ribosome additionally showed significant positive within‐complex effects (FDR‐adjusted *q* = 0.0033), whereas it was not significant when all annotated subunits were considered. The remaining significant complexes were unchanged.

#### 2.3.2 Are within‐complex influences stronger than influences on non‐members?

Three of the eight complexes met the statistical requirements to satisfy this query: Nop56p‐associated pre‐rRNA (adjusted $p=5.2\times 10^{-10}$), the cytoplasmic ribosome (adjusted $p=5.3\times 10^{-8}$), and the PA700‐20S‐PA28 complex (adjusted p=0.0176). These complexes display stronger internal effects compared to their influence on protein abundance outside the complex. When restricting the analysis to inferred core‐like proteins, the same three complexes remained significant, and the PA700 complex additionally satisfied this criterion (adjusted p=0.0214).

#### 2.3.3 Do complex members act in a consistent direction on outside targets?

Investigating this, the most widespread patterns of coordination among complexes were revealed. Each of the eight initially selected complexes showed significant directional concordance toward external targets, thus allowing us to extend this analysis to all CORUM complexes with three or more detected subunits, with 956 complexes in total; see Figure 2d and refer to the Supplementary Information for more detailed results. Large complexes with dense connectivity, such as the cytoplasmic ribosome, Nop56p‐associated pre‐rRNA complex, spliceosome A complex, and the nuclear pore complex, showed highly significant *q*‐values, reflecting the statistical power gained from including more outside targets available for testing. However, their median sign‐concordance values were modest, with 0.081 for the cytoplasmic ribosome, 0.133 for the Nop56p‐associated pre‐RNA complex, 0.2 for spliceosome A, and 0.334 for the nuclear pore complex.

Notably, several complexes displayed median concordance values of 0, such as NADPH oxidase, suggesting an equally mixed effect toward outside targets. On the contrary, however, 57 complexes showed perfect concordance, with a median of 1.0. Among those scoring perfect concordance, 30 complexes achieved statistically significant FDR adjusted *q*‐values, reflecting strong uniform agreement in the direction of influence across the outside genes. These complexes span diverse biological roles, including mitochondrial import and carrier systems, such as with the TIM22 and TOM complexes, DNA repair and genome maintenance, such as with the NEIL1‐PNKP‐Polβ‐LIG3‐XRCC1 complex, and endosomal and vesicle trafficking regulators, such as with GAIT and EARP complexes. The full list of the 30 complexes can be found in Supplementary Information. These fully concordant complexes, made up of between three and seven subunits, impose a unified “push” on their proteomic targets.

### 2.4 Pathway‐level regulatory structure

Then, it was investigated whether a similar coordinated pattern exists at the pathway level. Using KEGG pathway annotations, it was examined whether genes within the same pathway tend to influence each other's protein abundance in a consistent direction. For each pathway, all coefficients between pairs of pathway members were extracted, and the within‐pathway patterns of the eight most populated ones were visualized; see Supplementary Figure S3. The eight KEGG pathways that were investigated displayed heterogeneous regulatory patterns, with a mixture of positive and negative within‐pathway coefficients whose proportions vacillate around a range of 50%–60% in either direction. Overall, it can be gleaned that pathway membership does not imply uniform directionality of regulatory influence among genes.

### 2.5 Cancer hallmark gene influence

Moreover, we investigated whether genes linked to classic cancer hallmark terms (Hanahan & Weinberg, 2000; Hanahan & Weinberg, 2011) have higher influence scores than genes not associated with any hallmark. For each hallmark, we calculated an influence score for every gene, defined as the mean absolute coefficient across all other models (excluding self), and compared the distribution of hallmark genes with that of the background consisting of all genes not associated with any hallmark. The hallmarks assessed were sustaining proliferative signaling and evading growth suppressors, resisting cell death, enabling replicative immortality, inducing angiogenesis, activating invasion and metastasis, genome instability, and deregulation of cellular energetics; further detailed gene lists are featured in Supplementary Table S1. Additionally, a pooled “Union of 7 Hallmarks” set was defined, containing all genes annotated with at least one hallmark.

**FIGURE 3:**
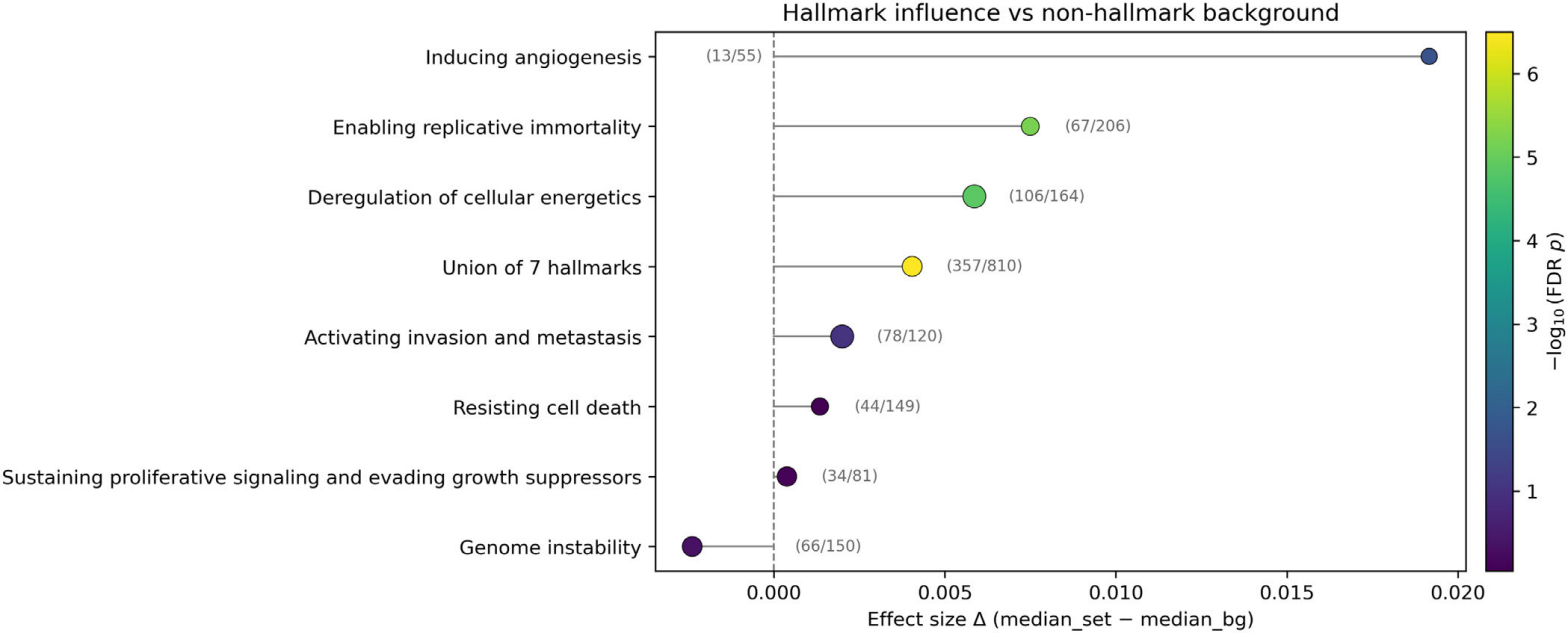
Genes of some cancer hallmarks show elevated regulatory influence in transcript‐protein models. For each hallmark, as well as for (and the pooled union, the “lollipop” markers in the graph show the difference in median influence score between hallmark genes and a non‐hallmark background. Circle size reflects the fraction of hallmark genes represented in our modeling universe, as written to the right of the circles in the parentheses. Circle color indicates statistical significance, as displayed by the gradient of significance scores on the right.

Three individual hallmarks, namely enabling replicative immortality, deregulation of cellular energetics, and inducing angiogenesis, showed significantly higher influence scores than the non‐hallmark background set such that FDR< 0.05, as did the pooled union set; see Figure 3. These results indicate that genes associated with certain hallmark programs tend to, on average, exert more polarized directional influence on the proteome in cancer patients, whereas others present no strong deviation from background levels.

To evaluate whether the observed hallmark influence could simply be explained by network connectivity, we quantified the number of STRING interaction partners for hallmark and background genes (Figure S4). While several hallmark categories contained genes with higher interaction counts than the background, influence scores were not positively associated with the number of interaction partners across all genes (Spearman *ρ* = −0.075). Moreover, the hallmark with the largest observed influence effect, inducing angiogenesis, had fewer interaction partners than the non‐hallmark background (median 6 vs. 10). These results suggest that the increased influence of hallmark genes is unlikely to be explained solely by them having higher network connectivity.

### 2.6 Latent structure in coefficient space

**FIGURE 4:**
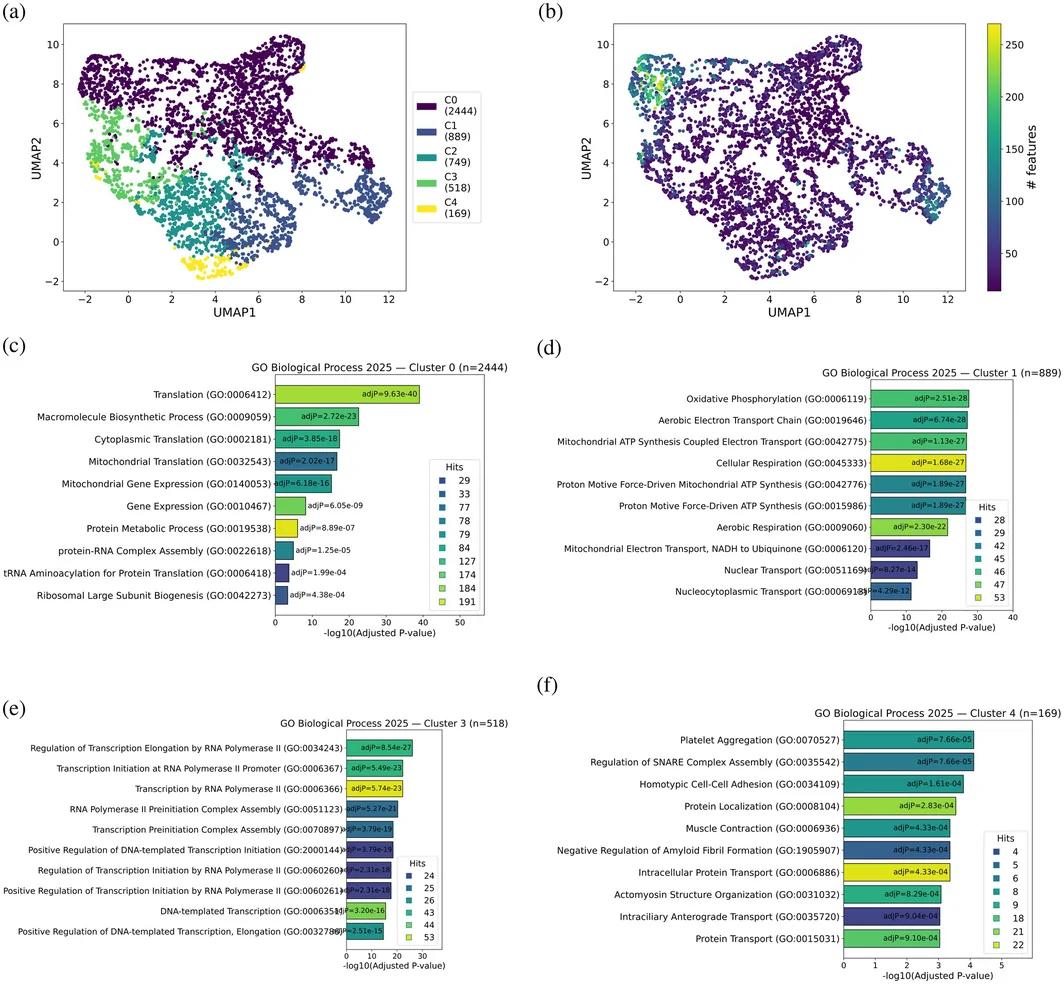
Autoencoder‐derived latent representations reveal functional organization of gene‐level models. (a) UMAP (McInnes et al., 2018) projection of the latent space, showing five distinct clusters (C0–C4) identified by hierarchical clustering. (b) UMAP colored by the number of transcript features included in each model. (c)–(f), Gene Ontology (GO) detailing Biological Process enrichment for genes within clusters C0, C1, C3, and C4, respectively.

Finally, it was investigated whether genes with similar linear coefficient profiles share common functional roles. For this, the linear models of each gene were expanded by including all non‐interactions with zero weight coefficients. Hence, when comparing the 5447 element vectors of two genes, their *n*th element always refers to the same cellular mRNA level. An autoencoder was trained that maps these input vectors to themselves and reduces the dimensions of the input vector to a 25‐dimensional transformer plane before decoding. Using the autoencoder‐derived latent embeddings of our model coefficients, hierarchical clustering was applied to group genes with similar regulatory signatures; then, Biological Process enrichment was evaluated for each cluster. The 25‐dimensional embeddings of the autoencoder were also projected into UMAP‐space for visualization of the models, which were colored by the respective clusters they belonged to; see Figure 4a.

As visualized in Figure 4b, it was assessed whether the clustering was simply driven by the number of interaction features available for each gene model; however, coloring the UMAP with respect to feature count showed no strong separation by model size, implying that the latent space captures higher‐order structure beyond the quantity of features each model possessed.

This analysis revealed five major clusters, dubbed C0–C4, that possess varying sizes, with one large cluster containing 2444 genes, while the others ranged from ~150 to ~900 genes in size, indicating both broad and more specialized groups. To evaluate whether these clusters reflected meaningful biological structure, GO Biological Process enrichment was performed for each cluster; see Figure 4c–f. Four of the five clusters exhibited significant enrichment for coherent functional themes.

Cluster 0, the largest group, was strongly enriched for translation and ribosome biogenesis processes, suggesting that genes involved in protein synthesis share similar transcript‐protein relationships, with their coefficients reflecting the tight co‐regulation of translational machinery. Then, it was determined that cluster 1 was associated with mitochondrial respiration, including terms such as aerobic electron transport chain, mitochondrial ATP synthesis, aerobic respiration, and oxidative phosphorylation, indicating that energy‐production modules are captured as a distinct regulatory signature in the latent space. Next, cluster 3 was correlated with transcriptional regulation, showing enrichment for transcription initiation, regulation of transcription initiation, and transcription elongation. In particular, this cluster contains several transcription factors and RNA polymerase subunits, whose proteomic abundances depend on a combination of transcriptional activity and post‐translational control; their embeddings being consolidated into a single cluster suggests that the autoencoder captured this shared mode of regulation. Finally, Cluster 4 was enriched for protein transport, protein localization, and cytoskeletal organization. This cluster contains genes with heterogeneous functions; their co‐clustering in latent space may indicate a shared regulatory mechanism linked to cellular structure and motility, which are key processes that shape cancer cell morphology and invasion. However, C2, the remaining cluster, showed no such significant enrichment, with further detail being provided in Supplementary Figure S5.

Interestingly, when analyzing the raw regression coefficients directly, a consistent pattern was observed such that in particular, the set of interactor‐driven genes described in Methods Section 4.3.4 was significantly enriched for processes related to translation and mitochondrial respiration; see Supplementary Figure S6. These enrichment patterns strongly overlap with those seen in clusters 0 and 1, reinforcing that the autoencoder captured biologically meaningful structure already present in the raw model coefficients. In contrast, the self‐driven genes did not show significant enrichment for any GO biological process after multiple testing correction (Supplementary Figure S7). This suggests that self‐driven genes are functionally more heterogeneous, whereas interactor‐driven genes are concentrated in coordinated processes such as translation and mitochondrial respiration.

## 3 DISCUSSION

In this body of work, explicit gene‐level interaction‐aware models were implemented across 10 various cancer types to quantify how each transcript level contributes to the abundance of each protein, allowing for the examination of regulatory influence at a resolution that is typically latent. By analyzing the structure of the learned coefficients, it can be assessed whether these inferred influences mirror known biological organization and uncover novel patterns of proteome‐level coordination. The results reveal several consistent patterns, both at the level of individual genes and at the level of higher‐order structure, such as complexes, pathways, and functional groups, which offer a concerted, mechanistic view of how proteomic abundance is shaped in tumors.

Our models captured transcript‐protein relationships with a median Pearson correlation of 0.64 across 5447 genes. The dominant contributor in most models was the gene's own transcript, with approximately 80% of gene models bearing a self‐coefficient exceeding all other interactor coefficients. The drop in performance that occurs when the self‐transcript is excluded from the prediction indicates that while interaction features capture complementary information, the gene's own transcript remains the sole strongest predictor of its proteomic abundance. This pattern is expected, as a substantial portion of protein variation reflects transcript abundance directly. At the same time, a subset of genes proved to be interactor‐driven, relying more strongly on the transcripts of their interaction partners than on their own. These genes exhibited distinct molecular and functional properties compared with self‐driven genes, including weaker transcript‐protein coupling, longer RNA half‐lives, and enrichment for coordinated processes related to translation and mitochondrial respiration.

An important question raised by these findings is why some genes are predominantly self‐driven, whereas others depend more strongly on the transcripts of their interaction partners. One molecular property distinguishing the two groups was RNA stability: interactor‐driven genes had significantly longer‐lived transcripts than self‐driven genes (Section 2.2 and Supplementary Figure S8a). One possible interpretation is that greater transcript stability reduces the dependence of protein abundance on the instantaneous abundance of the gene's own transcript and allows coordinated regulatory signals associated with interaction partners to become comparatively more informative. This interpretation is consistent with the enrichment of interactor‐driven genes for translation and mitochondrial respiration, both of which involve coordinated, multicomponent systems.

Previous studies have reported that long‐lived proteins generally display stronger mRNA‐protein correlations (Li et al., 2021; Wu et al., 2008), whereas mRNA half‐life was found to contribute relatively little to mRNA‐protein correlation in yeast (Wu et al., 2008). Our results address a related but distinct question. Rather than establishing RNA half‐life as a general determinant of mRNA‐protein correlation, they show that RNA half‐life differs between genes whose protein abundances are predominantly associated with their own transcripts and those whose protein abundances are more strongly associated with the transcripts of interaction partners. This association does not establish a causal mechanism, but it identifies RNA stability as a molecular feature that distinguishes the two regulatory model structures.

The comparison to random‐feature baselines further clarifies the contribution of interaction‐aware information. When the gene's own transcript was included, the difference between interactor‐based and random models was negligible, highlighting the indispensable influence of the self‐transcript. However, when the self‐transcript feature was removed, models using STRING interactors noticeably outperformed those using random genes, indicating that interaction‐based predictors carry biological signal that becomes more apparent once the contribution of the self‐transcript is removed.

The present framework specifically evaluates transcript levels of genes whose encoded proteins are functionally associated according to STRING. Other regulatory layers, such as transcription factor‐target gene relationships, were not included because they represent a distinct mode of regulation acting primarily through DNA rather than protein–protein or protein‐mRNA interactions. Besides, previous work has shown that transcription factor and target‐gene expression levels are often only weakly correlated on average (Hughes & de Boer, 2013). Incorporating transcriptional regulatory networks alongside protein interaction networks therefore represents an interesting direction for future work.

The implementation of linear regression models was carried out for several reasons. First, linear models are more intuitive to train and interpret because the learned coefficients directly quantify the direction and magnitude of each feature's influence on the target protein. In fact, since standardization was applied to the features, each coefficient can be interpreted as the expected change in protein expression for a one‐standard‐deviation increase in the corresponding feature, when keeping the other variables constant. Furthermore, each coefficient in a linear regression model is accompanied by statistical measures such as p‐values and bootstrap 95% confidence intervals. These provide an assessment of coefficient significance and stability, allowing the robustness of individual transcript‐protein relationships to be evaluated. In contrast, while random forests can produce feature importance scores, these are relative and non‐directional, making this method less suited for constructing interpretable influence networks. Moreover, although random forest models produced similar distinctions between interactor‐based and random predictors, their predictive accuracy remained lower than that of linear models.

One of the clearest examples of coordinated behavior exemplified in our models is that of the cytoplasmic ribosome, which is the most abundant and one of the largest complexes in the eukaryotic cell (Beck et al., 2011); thus, it is sensible for the cell to synthesize protein components in strictly stoichiometric proportions. In contrast to most other protein complexes, whose members exhibit strong self‐driven behavior, as illustrated by the bright red diagonal in Figure 2b,c, ribosomal genes showed almost no diagonal dominance in their coefficient heatmap, as displayed in Figure 2a; ribosomal self‐coefficients were weak and the predictive signal was instead distributed across the incoming interactor coefficients. Supporting this observation, transcript‐protein Pearson correlations for ribosomal genes in the dataset were uniformly low, ranging from −0.12 to 0.4 across the 68 subunits considered, indicating that protein abundances of individual ribosomal components are not well reflected by their own transcript levels. To evaluate whether this coordinated behavior could simply reflect the large number of predictors included in the ribosomal models, we repeated the analysis after separating the cytoplasmic ribosome into its 40S and 60S subunits and restricting predictors to interactors within the corresponding subunit. This reduced the median number of interactors from 211 to 28 for 40S genes and from 171 to 40 for 60S genes. Despite this substantial reduction in model complexity, 52 of the 58 originally interactor‐driven ribosomal genes remained interactor‐driven, while self‐coefficients increased only modestly (median increase 6.9%). These findings indicate that the characteristic coefficient pattern of ribosomal genes is robust to the number of predictors included in the models.

This conclusion was further supported by comparing the cytoplasmic ribosome with the five largest non‐ribosomal CORUM complexes. If the observed behavior were primarily explained by complex size, other complexes of similar size would be expected to show comparable enrichment for interactor‐driven genes. However, this was not observed. For example, only 23 of 110 genes (20%) in the Spliceosome E complex were interactor‐driven, compared with 58 of 68 (85%) ribosomal genes, whereas the mitochondrial ribosome and respiratory chain complex I showed proportions more similar to those of the cytoplasmic ribosome. These findings further suggest that the ribosomal pattern cannot be explained solely by model size.

To assess whether this behavior is unique to the ribosome or shared by other highly abundant protein complexes, the 26S proteasome, the second‐most abundant protein complex in human cells, was examined. The corresponding coefficient patterns of the 26S proteasome showed an intermediate pattern between that of the ribosome and other complexes, with self‐coefficients generally weak but not as uniformly diminished as the ribosome; see Supplementary Figure S9. Notably, ribosomal transcripts also exhibited substantially longer RNA half‐lives, at a median length of 16.8 h, than those of the spliceosome and nuclear pore complex (NPC), at a median of 3.8 h ($p=1.0\times 10^{-29}$). Such prolonged RNA stability provides a plausible explanation for the ribosome's diffuse coefficient structure; when transcripts persist for many hours, extant protein levels may depend less on instantaneous self‐transcript abundances and more on coordinated regulation from other subunits. This interpretation aligns with our aforementioned finding that interactor‐driven genes are enriched for translational processes, where stoichiometric balance and coordinated assembly impose collective regulation; see Supplementary Figure S6. The RNA half‐life distribution of ribosomal transcripts and those of the spliceosome and nuclear pore complex is provided in Supplementary Figure S8b.

Interestingly, some members of certain protein complexes have a strictly positive or negative effect on all other constituents, as seen in the RANBP2 gene that encodes Nucleoporin 358, the largest nucleoporin located on the cytoplasmic filaments of the Nuclear Pore Complex (Desgraupes & Arhel, 2025). This gene appears as a uniformly blue column in Figure 2a, indicating that its transcript contributes negatively to the predicted protein abundance of the other NPC subunits.

Ultimately, how transcript levels of a certain gene impact protein abundances of another gene must be further elucidated. It is highly unlikely that other transcripts physically interact with mRNA in the cell. Instead, we suggest an indirect mechanism for this effect: it has been frequently reported that proteins possibly bind to their own mRNA transcript (Kapral et al., 2022). However, comparison with the currently available set of experimentally validated autogenous RNA‐binding proteins did not reveal a significant difference between self‐driven and interactor‐driven genes, although the limited number of validated proteins precludes firm conclusions. Possibly, at the same time, other protein interactors could be bound to these proteins that interact with their own transcripts, hindering accessibility to their own respective mRNA. Given that these protein interactors also bind to their own respective transcripts and that their abundances are determined by their transcript abundances, it is possible that these protein interactor mRNA transcripts could indirectly affect the accessibility of other transcripts to be translated, the proteins of which interact with these interactors. Thus, between proteins and their protein interactors, as well as with their respective self‐transcripts, there may exist an equilibrium between transcriptional abundances and protein abundances due to this competitive binding.Of course, many more factors are relevant when relating mRNA to protein levels (Liu et al., 2016; Maier et al., 2009). For example, it was shown that while total mRNA levels in a cell can vary significantly as a function of cellular state (Marguerat et al., 2012), protein levels generally vary in a much smaller window. Furthermore, tumors often show genomic copy number alterations of genes. Whereas both mRNA and protein levels were generally positively correlated with such copy number changes, many copy number alterations leading to mRNA changes did not translate into corresponding protein level changes in tumors (Zhang et al., 2014).

In conclusion, this study defines two distinct classes of genes, on a continuum: “self‐driven” genes, which are relatively short‐lived genes whose protein abundances are directly proportional to their own mRNA abundances with an average Pearson correlation of 0.58, and “interactor‐driven” genes, which are longer‐lived genes whose protein abundances are more influenced by the transcript levels of other genes, mainly genes that encode protein–protein interactors, as opposed to random genes. These insights suggest several clinical implications. In modern omics, diagnostic criteria of disease often include the transcription levels of certain biomarker genes. For self‐driven genes, interpretation of biomarker levels is far more intuitive than that of interaction‐driven genes, posing a novel challenge for further defining explicit diagnostics. These findings can also be applied to therapeutic treatments that aim to manipulate protein abundance levels, as tuning protein abundances of an interactor‐driven gene is far more complex than for self‐driven genes.

## 4 METHODS

### 4.1 Datasets

#### 4.1.1 Data acquisition and pre‐processing

Transcriptomic and proteomic data were obtained from the Clinical Proteomic Tumor Analysis Consortium (CPTAC) via the cptac Python interface (https://pypi.org/project/cptac/). Paired profiles were retrieved from the Baylor College of Medicine (BCM) datasets for the 10 cancer types examined: breast cancer (BRCA, *n* = 121), clear cell renal cell carcinoma (CCRCC, *n* = 175), colon adenocarcinoma (COAD, *n* = 96), glioblastoma (GBM, *n* = 99), head and neck squamous cell carcinoma (HNSCC, *n* = 154), lung squamous cell carcinoma (LSCC, *n* = 202), lung adenocarcinoma (LUAD, *n* = 207), ovarian carcinoma (OV, *n* = 82), pancreatic adenocarcinoma (PDAC, *n* = 120), and uterine corpus endometrial carcinoma (UCEC, *n* = 95). Only patients with both transcriptomic and proteomic data were retained, yielding a total of 1351 matched samples, including 328 derived from normal tissue.

Then, the analysis was further restricted to genes possessing both transcriptomic and proteomic measurements available across all cancer types, resulting in 7782 common genes. Genes possessing over 30% missing values across transcriptomic and proteomic features were removed, leaving a final total of 6054 genes for analysis. Remaining missing values were imputed using K‐nearest neighbors imputation, using the KNNImputer implementation in scikit‐learn v1.3.0 (Pedregosa et al., 2011) with a *k* = 10, applied separately within each cancer type.

During the early stages of investigation, the PPIXpress (Will & Helms, 2016) software was used to define interactor partners for each gene. PPIXpress constructs condition‐specific protein–protein interaction networks by integrating transcriptomic and proteomic profiles for each sample. From these networks, each gene's interactors can be extracted as model features. Due to domain annotation and mapping requirements within PPIXpress, this step yielded a subset of the total genes available, resulting in a working set of 5447 genes.

Initially, our models were trained using these derived PPIXpress interactors and subsequently, model performance was compared to random‐feature models. In these models, the performances of interactor‐based and random predictor models were nearly identical when the gene's self‐transcript was included, with only a modest improvement achieved upon excluding self‐transcripts; see Supplementary Figure S10. To further evaluate the role of interaction information, the STRING database (v12.0) (Szklarczyk et al., 2019) was implemented as an alternative source of interactors. Specifically, the STRING 800 interactors were selected over STRING 200 and CORUM (Giurgiu et al., 2019) interactors due to the result seen in Srivastava et al. (2022). For a fair comparison with PPIXpress, the same 5447 genes were retained. After comparing the two approaches, the study was ultimately carried out with STRING‐defined interactors for all subsequent modeling and analyses.

#### 4.1.2 Model input files

**FIGURE 5:**
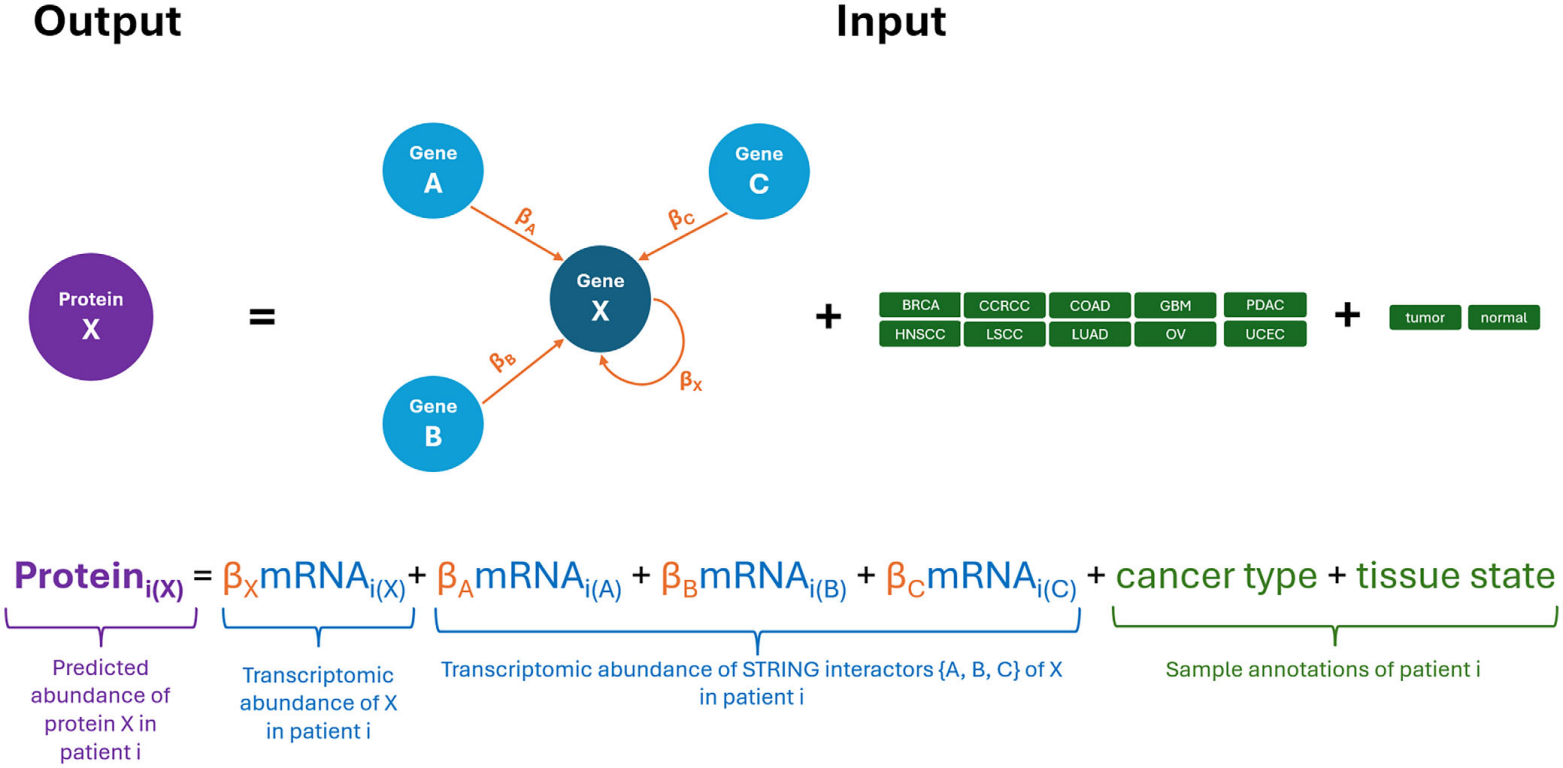
Linear Regression predictive model: Schematic of the linear regression model predicting each gene's protein abundance from its own transcript, transcripts of known STRING interactors, and categorical features.

For each of the 5447 retained genes, a separate input file was created for regression modeling. Each row of the file contained the gene's own transcript expression level in each of the 1351 samples, together with the transcript expressions of its interaction partners in the same sample; interaction partners were defined using the STRING database v12.0 at a combined score threshold of 800, and the corresponding protein abundance for the gene in the sample. Each file was further annotated with sample metadata: a cancer_type column indicating the cancer type for each sample, as well as a binary disease_status column, listed as “tissue state” in Figure 5, denoting tumor and healthy tissue as 1 and 0, respectively.

To establish a baseline for comparison, “random” input files were generated for each of the 5447 gene‐specific files. In these files, the target gene's own transcript expression, protein abundance, and sample metadata columns were retained. However, as opposed to STRING‐derived interactors, an equal number of genes was randomly selected from the 5447‐gene set and their transcript expression values were included as predictors. For the comparison shown in Figure 1c, five independent random sets were generated using different random seeds. To further assess the robustness of the comparison, this procedure was extended to 100 random sets per gene. The performance of each STRING‐based model was then compared with the mean performance of its 100 corresponding random models across genes using a one‐sided Wilcoxon signed‐rank test.

### 4.2 Linear regression models

One linear regression model was trained per gene (*n* = 5447). its proteomic abundance across all 1351 samples served as the response variable. Predictors consisted of the target gene's transcript expression, the transcript expression levels of its STRING interaction partners, cancer_type, and binary disease_status features. Categorical variables were one‐hot encoded, and continuous variables were standardized to have a mean of zero and a standard deviation of one. Division by a standard deviation of zero could only occur if a gene showed constant expression across all 1351 samples, which was not the case. Two predictor configurations were therefore evaluated: one including the target gene's own transcript and one excluding it.

Linear regression was performed using scikit‐learn v1.3.0 pipelines (Pedregosa et al., 2011) consisting of a StandardScaler, followed by LinearRegression; see Figure 5. For each gene, five‐fold cross‐validation was performed using cross_val_predict, ensuring that scaling and encoding were fit within each fold to prevent data leakage. Model performance was measured by the Pearson correlation coefficient between observed and predicted proteomic abundances. After performing cross‐validation, each model was refitted on the full dataset to obtain the resulting regression coefficients.

To establish a baseline, random models were trained using the random gene‐selection input files while retaining the corresponding transcript abundance profiles and were evaluated in the same manner as the interaction‐based models.

To assess the statistical significance of the regression coefficients, *p*‐values were computed post hoc under classical ordinary least squares assumptions. Following model fitting, predictors were standardized using the same scaling parameters as in the trained pipeline, and an intercept term was included. For each coefficient, standard errors were estimated from the residual variance and the inverse of the design matrix, and two‐sided *t*‐tests were used to calculate *p*‐values.

To assess coefficient uncertainty and stability, bootstrap 95% confidence intervals were computed for each coefficient. For every gene‐specific model, 1000 bootstrap samples were generated by resampling the 1351 observations with replacement using the choice method of NumPy's random. Generator. A fixed random seed of 42 was used to ensure reproducibility of the complete sequence of bootstrap samples. The complete modeling pipeline, including feature standardization and linear regression fitting, was refitted on each bootstrap sample using the same predictor set, and the resulting coefficient estimates were recorded. The lower and upper confidence limits were defined as the 2.5th and 97.5th percentiles of the bootstrap coefficient distributions, respectively. A coefficient was considered sign‐stable when its bootstrap confidence interval remained entirely above or entirely below zero.

To evaluate multicollinearity among predictors, variance inflation factors (VIFs) were computed as a diagnostic for each gene‐specific model. For a given predictor, the VIF quantifies the extent to which its variance is inflated due to linear dependence with the remaining predictors and was calculated by regressing each predictor against all other predictors in the same model. VIFs were computed using the standardized design matrix and were restricted to continuous transcript features, excluding one‐hot encoded categorical covariates because these can introduce structural collinearity and non‐informative infinite VIF values. Models with fewer than two continuous transcript predictors were excluded because VIF is undefined in this setting.

### 4.3 Coefficient analyses

To quantify how genes influence each other's expression patterns at the protein level, the regression coefficients were analyzed from the per‐gene models. For each target gene, its model predicts protein abundance from its own self‐transcript and the transcripts of its interaction partners. The coefficients of models that include the gene's own self‐transcript were extracted as a feature during training, then excluded during analysis; all remaining coefficients were treated as the gene's outgoing influence on the network. Genes that only appeared as predictors in their own model, such that they possessed no STRING interactors in the gene set, were excluded from the analysis, resulting in a total of 4769 genes, which were dubbed as the “analysis universe”.

Coefficients were effectively treated as measures of influence that a gene's transcript abundance has on the proteomic abundance of another gene. Because expression values were mean‐centered and scaled, each coefficient reflects the change, in standard deviation units, of a gene's protein abundance associated with a one standard deviation change in another gene's transcript abundance. In terms of defining “influence”, the mean absolute value of coefficients that a gene's own transcript has across all models in which it appeared as a predictor for other genes, excluding its own model, was used.

#### 4.3.1 Protein complex influence analysis

Subsequently, it was assessed whether members of annotated protein complexes act in a coordinated manner. Human complexes were obtained from the CORUM database, in the “Human Complexes” release (https://mips.helmholtz-muenchen.de/corum/). For each complex, its annotated subunits were intersected with the analysis universe, retaining complexes with at least three subunits present. For these complexes, coverage, defined as the fraction of subunits present, and the number of within‐complex edges, defined as coefficients between pairs of complex members available in our models, were recorded.

Thus, three properties were evaluated. First, positive within‐complex regulation was assessed: for each complex, the median of the signed coefficients incoming from other complex members was computed for each member gene. The resulting per‐gene medians were tested against zero using a one‐sided Wilcoxon signed‐rank test; upon demonstrating statistical significance, the alternative hypothesis asserts that the median within‐complex coefficient is greater than zero, indicating predominantly positive regulation among members. Second, the magnitude of within versus outside effects was evaluated: for each gene in the complex, the median absolute value of coefficients from other “within” complex members was compared to those from “outside” non‐member predictors. These paired values were evaluated against a paired, one‐sided Wilcoxon signed‐rank test, with the alternative hypothesis stating that within‐complex coefficients have greater magnitude than outside‐complex coefficients. Third, directional agreement toward outside targets was estimated: to assess whether complex members act in a consistent direction on genes outside of the complex, for each outside target, a sign‐concordance metric was computed. For each outside target *k*, {*β*_*i*‐>k_} serves as the member‐to‐target coefficient; at least 3 members of the complex were required to have non‐zero coefficients for that target. Then, the sign‐concordance metric was computed per target:

$$c_{k}=\left|\text{mean}\left(\mathrm{sign}\left(\beta_{i->k}\right)\right)\right|\in \left[0,1\right]$$

A value of *c*_*k*_ = 1 signifies that all constituents within the complex act on the outside target in a uniform direction, either in an all‐positive or all‐negative manner, whereas a value near 0 indicates no directional agreement due to offsetting positive and negative coefficients. For each complex, this agreement was summarized by the median *c*_*k*_ across outside targets and tested using a one‐sided Wilcoxon signed‐rank test, with the alternative hypothesis asserting that the median is greater than zero. All resulting *p*‐values were corrected for multiple testing across complexes using the Benjamini–Hochberg false discovery rate procedure.

To evaluate whether coordinated behavior might be masked by averaging across functionally distinct complex members, a secondary analysis was performed using only inferred core‐like proteins. As no comprehensive annotations distinguishing core/scaffold and peripheral subunits were available for the human CORUM complexes analyzed in this study, a co‐expression‐based proxy was adopted following the core/halo concept proposed by Dezso et al. (2003) and later discussed for mammalian protein complexes by Steinkamp et al. (2025).

For each analyzed complex, pairwise Pearson correlations between transcript abundances were computed across all 1351 samples. For every protein, the mean transcript co‐expression with all other members of its complex was calculated. Following Dezső et al., proteins were classified as core‐like if their mean co‐expression exceeded the complex‐specific threshold of the mean minus one standard deviation; the remaining proteins were classified as halo‐like.

The analyses of positive within‐complex regulation and within‐ versus outside‐complex coefficient magnitude described above were then repeated after restricting each complex to its inferred core‐like proteins.

#### 4.3.2 Pathway‐level within‐pathway structure

To examine pathway structure, pathway annotations were procured from the MSigDB (Liberzon et al., 2011) KEGG legacy collection v2025.1 and used for pathway analysis. For each KEGG pathway, its member genes were intersected with the analysis universe, quantifying the number of overlapping genes and the number of “within‐pathway edges,” defined as available coefficients where both predictor and target belong to the same pathway. Pathways were ranked by the number of overlapping genes, with the eight most populated pathways being selected for visualization.

For each selected pathway, a square coefficient matrix was constructed, featuring rows and columns listing the member genes of the pathway that appeared both as regression targets and as predictors. Off‐diagonal entries represent the effect of one pathway member's transcript on another member's protein abundance. For visualization, each gene's own self coefficient was overlaid on the diagonal. These heatmaps were considered to assess whether pathway members tend to up‐ or down‐regulate each other and whether regulatory structure appears coordinated.

#### 4.3.3 Hallmark‐of‐cancer influence analysis

It was then queried whether genes linked to canonical hallmarks of cancer tend to exert stronger influences on protein abundances than other genes within the analysis universe. Seven curated hallmarks were used: “sustaining proliferative signaling,” “evading growth suppressors,” “resisting cell death,” “enabling replicative immortality,” “inducing angiogenesis,” “activating invasion and metastasis,” and “genome instability and mutation”; hallmark gene sets are provided in Supplementary Table S1.

For each hallmark, its gene set was intersected with the analysis universe and the distribution of mean absolute coefficients was compared across all other models, excluding self models, for hallmark genes to a background consisting of all genes not assigned to any of the seven hallmarks (“non‐hallmark” background); this same background was used for all comparisons. Distributions were compared using a two‐sided Mann–Whitney U test. For each hallmark, the median influence in the hallmark set, the median influence in the background, and the difference (median_set *−* median_bg) between the two were reported as an effect‐size summary.

The same comparison was repeated for the pooled union of all genes appearing in any of the seven hallmarks, titled “UNION_OF_7_HALLMARKS”, against the same non‐hallmark background. Raw *p*‐values from all eight tests, including seven individual hallmarks in addition to the pooled union, were adjusted using the Benjamini‐Hochberg procedure to obtain FDR values.

#### 4.3.4 Self‐ versus interactor‐driven models

For each gene‐specific model, the contribution of the gene's own transcript, as indicated by the self‐coefficient, as well as its STRING‐derived interactors, indicated by the interactor coefficients, was quantified. Coefficients were treated in absolute magnitude. A model was required to include at least three interactors in order to be considered for further analysis. A gene was defined as strongly interactor‐driven if the largest absolute interactor coefficient was at least twice the magnitude of the self‐coefficient and classified as strongly self‐driven if the absolute self‐coefficient was at least twice the sum of the absolute interactor coefficients.

#### 4.3.5 Functional enrichment analysis

Given a gene set of interest, such as high‐influence genes, strongly interactor‐dominant genes, or clustered genes, overrepresentation of GO Biological Process terms was tested using Enrichr (Chen et al., 2013) and the “GO Biological Process 2025” library for human genes. For each analysis, an explicit background was supplied, consisting of the 4769 genes in the analysis universe. Enrichment was evaluated using a Fisher's exact test; *p*‐values were adjusted for multiple testing using the Benjamini–Hochberg procedure (FDR).

#### 4.3.6 RNA half‐life analysis

RNA half‐life measurements were obtained from the compilation provided by RNAdecayCafe (Vock et al., 2025). The file AvgKdegs_genes_v1.csv, which reports per‐gene RNA degradation rates and half‐lives across multiple tumor cell lines, was used. For each gene, a cell‐line‐averaged, donor‐normalized half‐life was computed by grouping entries with respect to gene symbol (feature_ID) and taking the mean of the avg_donorm_halflife values.

To compare RNA stability across gene sets, statistical differences were assessed using the one‐sided Mann–Whitney U test, with the direction of the alternative hypothesis set according to the comparison of interest.

### 4.4 Autoencoder Embeddings analysis

**FIGURE 6:**
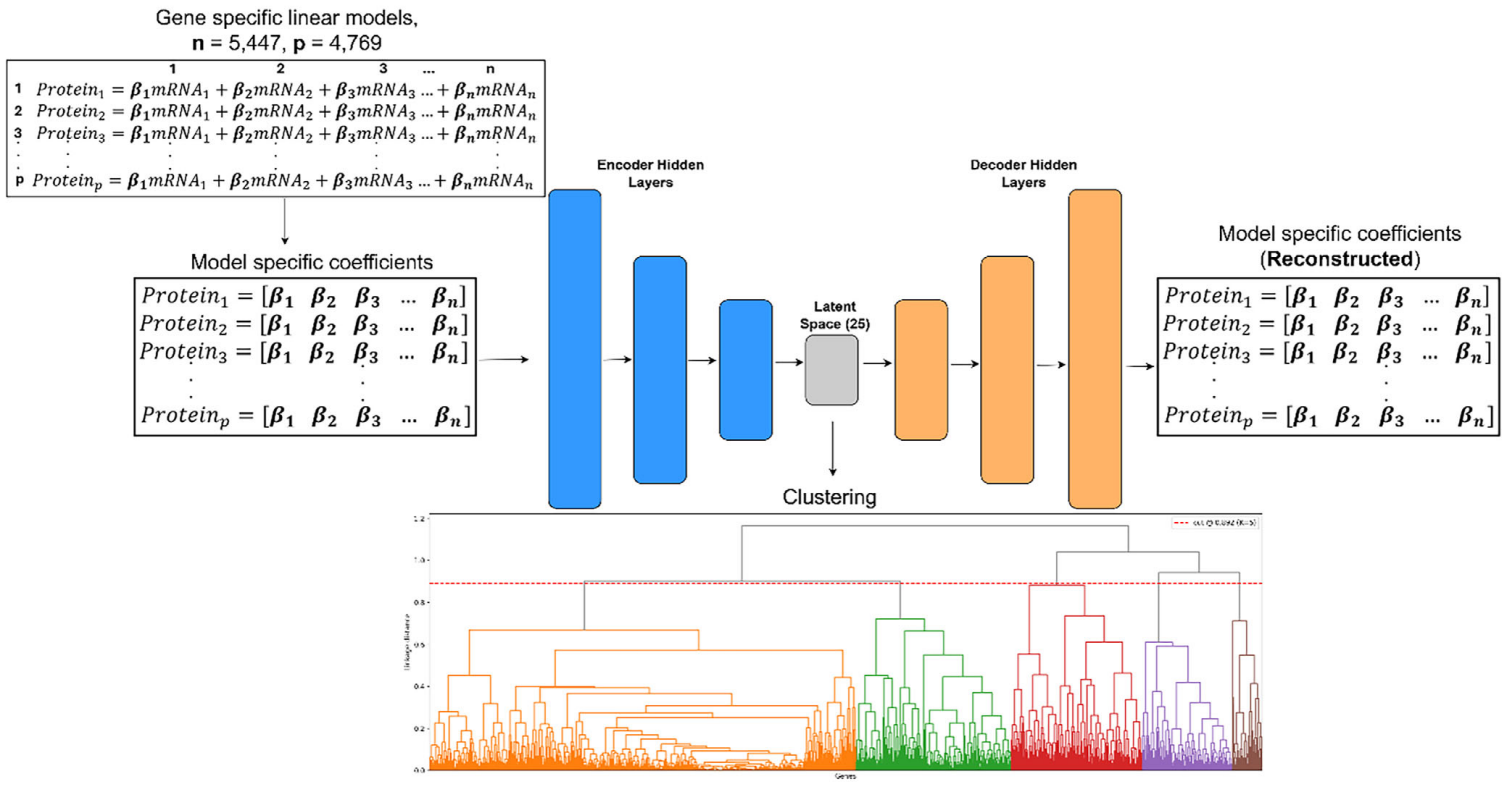
Autoencoder implementation and usage. The missing coefficients in each gene‐based model are initially replaced with zeros. Then, the coefficients are extracted and used as inputs to the autoencoder. The autoencoder reproduces these coefficients using the 25‐dimensional latent space embeddings. Clustering was performed based on the latent space embeddings for each gene's model coefficients.

Coefficient vectors from the 4769 analysis universe genes possessing at least one interactor were used as model representations. Missing coefficients were replaced with zeros to obtain an equal size vector for each gene model. An autoencoder was implemented using PyTorch v2.6 (Paszke et al., 2019). Training was performed for 10,000 epochs using mean squared error loss and the Adam optimizer, with a learning rate of 1e‐5 (Kingma & Ba, 2015). The trained autoencoder produced 25‐dimensional embeddings for these genes based on their model coefficients. Prior to clustering, these embeddings were standardized via Z‐scoring. Agglomerative hierarchical clustering was performed using the average linkage method and the correlation distance metric, as implemented in SciPy (Virtanen et al., 2020). The dendrogram was cut to yield *K* = 5 clusters. The complete workflow conducted for autoencoder creation and latent space clustering is illustrated in Figure 6. Uniform Manifold Approximation and Projection (UMAP) was applied to the standardized embeddings using the umap‐learn package for two‐dimensional visualization. The projection was computed with the parameters *n*_neighbors = 15, min_dist = 0.1, and the correlation distance metric. Genes were plotted in UMAP space and colored by their assigned hierarchical cluster or the number of features their model utilizes. For each cluster, GO Biological Process enrichment was performed using the methods outlined in Section 4.3.5, with the studied gene‐set taken into account as the background.

## AUTHOR CONTRIBUTIONS

**Aram Papazian:** Conceptualization; writing – review and editing; validation; methodology; supervision. **Loulwah Arnaout:** Conceptualization; investigation; writing – original draft; methodology; validation; visualization; formal analysis; data curation. **Volkhard Helms:** Conceptualization; writing – review and editing; validation; project administration; supervision.

## Supporting information


**Figure S1.** Distribution of VIF values computed for interactor predictors across all models, excluding categorical covariates; models with <1 interactor are removed.
**Figure S2.** Random Forest models using STRING800 interactors. Boxplots represent the performance of gene‐models using interactors versus random genes as predictors, with *n* = 5447 per box. Models were built using sklearn's RandomForestRegressor, with n_estimators = 100.
**Figure S3.** Mixed regulatory influences among members of KEGG pathways. (a) Within‐pathway coefficient matrices for the eight most populated KEGG pathways, defined by the largest absolute overlap with the analysis universe dataset of 4769 filtered genes; overlaps range from 78 to 118 genes, corresponding to approximately 30%–80% pathway coverage. Rows correspond to gene‐specific models, and columns correspond to transcript features. Colors denote the sign and magnitude of each coefficient. Diagonal entries represent self‐coefficients. (b) Proportion of positive and negative coefficients among genes within each pathway, with edge counts displayed in gray text to the right.
**Figure S4.** Distribution of STRING interaction partners among hallmark and non‐hallmark genes. Boxplots show the number of STRING interaction partners for genes belonging to each cancer hallmark and for the non‐hallmark background. Numbers above the boxes indicate the number of genes in each group, and numbers within the boxes indicate the median number of interaction partners.
**Figure S5.** Biological Process enrichment of genes in Cluster 2 of the autoencoder embeddings.
**Figure S6.** Biological Process enrichment of the gene set containing interactor‐driven genes.
**Figure S7.** Biological Process enrichment of the gene set containing self‐driven genes.
**Figure S8.** RNA half‐life comparisons between gene categories. (a) Strongly self‐driven versus strongly interactor‐driven genes. (b) Ribosomal genes compared to nuclear pore complex (NPC) and spliceosome genes.
**Figure S9.** Within‐complex coefficient matrix for the 26S proteasome.
**Figure S10.** Linear regression models using PPIXPRESS interactors. Boxplots represent the performance of gene‐models using interactors versus random genes as predictors, with *n* = 5447 per box. The boxplots in blue show models that include the gene's own self‐transcript in the prediction, while the orange boxes show models that exclude it. The lighter‐colored boxes correspond to models built with random genes. Within the boxes, the red marks, along with the numbers displayed to the right of the boxes, show median values.
**Table S1:** Lists of genes associated with hallmarks of cancer.

## Data Availability

The processed transcriptomic and proteomic data used in this study, together with the code and trained regression models, are available on Zenodo (DOI: 10.5281/zenodo.21417532). The original CPTAC data are publicly available through the CPTAC Data Portal and can also be accessed using the cptac Python package. Details on data preprocessing and model input generation are provided in the repository and in the Methods section.
