# Supplementary material for: Relating transcriptomics to protein abundance reveals self‐driven versus interactor‐driven proteins
Source: Protein Sci. 2026 Sep 16;35(10):e70806. doi: 10.1002/pro.70806 (PMC13580347; doi:10.1002/pro.70806)
Supplement: Supplementary file 1 [file PRO-35-e70806-s001.docx]

**Figure S1: Distribution of VIF values computed for interactor predictors across all models,** excluding categorical covariates; models with <*1* interactor are removed.

**Figure S2:** **Random Forest models using STRING800 interactors.** Boxplots represent the performance of gene-models using interactors versus random genes as predictors, with n = 5,447 per box. Models were built using sklearn’s RandomForestRegressor, with n_estimators=100

**Figure S3: Mixed regulatory influences among members of KEGG pathways. a,** Within-pathway coefficient matrices for the eight most populated KEGG pathways, defined by the largest absolute overlap with the analysis universe dataset of 4,769 filtered genes; overlaps range from 78 to 118 genes, corresponding to approximately 30-80% pathway coverage. Rows correspond to gene-specific models, and columns correspond to transcript features. Colors denote the sign and magnitude of each coefficient. Diagonal entries represent self-coefficients. **b,** Proportion of positive and negative coefficients among genes within each pathway, with edge counts displayed in grey text to the right.

**Figure S4: Distribution of STRING interaction partners among hallmark and non-hallmark genes.** Boxplots show the number of STRING interaction partners for genes belonging to each cancer hallmark and for the non-hallmark background. Numbers above the boxes indicate the number of genes in each group, and numbers within the boxes indicate the median number of interaction partners.

**Figure S5: Biological Process enrichment of genes in Cluster 2 of the autoencoder embeddings.**

**Figure S6: Biological Process enrichment of the gene set containing interactor-driven genes.**

**Figure S7: Biological Process enrichment of the gene set containing self-driven genes.**

**Figure S8:** **RNA half-life comparisons between gene categories. a,** strongly self-driven versus strongly interactor-driven genes. **b,** ribosomal genes compared to nuclear pore complex (NPC) and spliceosome genes.

**Figure S9: Within-complex coefficient matrix for the 26S proteasome.**

**Figure S10: Linear regression models using PPIXPRESS interactors.** Boxplots represent the performance of gene-models using interactors versus random genes as predictors, with n = 5,447 per box. The boxplots in blue show models that include the gene’s own self-transcript in the prediction, while the orange boxes show models that exclude it. The lighter-colored boxes correspond to models built with random genes. Within the boxes, the red marks, along with the numbers displayed to the right of the boxes, show median values.

| Hallmark | Genes |
| --- | --- |
| Deregulation of cellular energetics | ALDOC, BPGM, ENO1, ENO2, ENO3, HK2, HK3, PFKL, PGK1, PGK2, PGM1, PGM2, PGM3, PKLR, TPI1, FBP1, FBP2, PC, PCK2, PDK1, PDK2, PDK3, PDPR, ACLY, ACO1, ACO2, CS, DLAT, DLD, DLST, FH, IDH1, IDH2, IDH3A, IDH3B, IDH3G, MDH2, OGDH, PDHA1, PDHB, SDHA, SDHB, SUCLA2, SUCLG1, SUCLG2, G6PD, H6PD, PGLS, PRPS2, RPE, RPIA, TALDO1, GBE1, GYS1, UGP2, AGL, PYGL, PYGM, GSK3A, GSK3B, PHKB, PHKG2, NDUFA10, NDUFA11, NDUFA2, NDUFA4, NDUFA5, NDUFA6, NDUFA8, NDUFB10, NDUFB3, NDUFB4, NDUFB5, NDUFB6, NDUFB7, NDUFB8, NDUFB9, NDUFC2, NDUFS1, NDUFS2, NDUFS3, NDUFS4, NDUFS5, NDUFS6, NDUFS7, NDUFS8, NDUFV1, NDUFV2, NDUFV3, CYC1, UQCRC1, UQCRC2, UQCRFS1, UQCRH, UQCRQ, COX4I1, COX5A, COX5B, COX6A1, COX6B1, COX6C, COX7A2L, COX7B, PPA1, DNAJB1, SLC25A25 |
| Activating invasion and metastasis | SMAD3, ITGB2, MAPK1, MYH10, PLAUR, PLD1, PRKCA, RAC2, WASF2, WIPF1, EGFR, IGF1R, ITGA4, ITGB1, MET, DPP4, EZR, MSN, ACTN1, ILK, MMP14, PTEN, PTK2B, PXN, RASA1, RHOA, ARHGEF7, BCAR1, CAPN1, CAPN2, CAV1, ENAH, MYL9, TLN1, VASP, VCL, AKT1, FAP, MMP2, MMP9, TIMP2, BAIAP2, CDC42, DIAPH1, RDX, CTTN, PIK3CA, WASL, ACTR2, ACTR3, ARF6, CFL1, SH3PXD2A, SRC, ARHGDIA, MYLK, PLCG1, PTPN1, RHOB, RHOC, VIM, CRK, PAK1, PAK4, RAC1, STAT3, PFN1, MMP7, CHD4, EWSR1, HTATIP2, MTA1, RB1, SMAD2, SMAD4, CTSL, CD82, METAP2 |
| Enabling replicative immortality | NBN, PARP1, PRKDC, PTGES3, RAD50, TEP1, TERF2, TERF2IP, XRCC5, XRCC6, GAR1, NHP2, NOP10, RAP1A, MSH3, AKT1, KRAS, MEN1, PPP2R1A, PPP2R1B, PRKCA, PRKCB, RB1, SART1, SMAD3, SP1, SSB, ATM, CDK4, CDK6, CHEK2, RBL2, ANG, IGFBP7, MMP14, TIMP1, TIMP2, PLAT, PLAU, CTSB, ICAM1, ICAM3, FAS, PLAUR, EGFR, FN1, LAMA2, LAMA4, LAMA5, LAMB1, LAMB2, COL6A1, COL6A2, COL6A3, COL12A1, COL14A1, COL16A1, COL1A1, COL1A2, COL3A1, COL5A1, COL5A2, COL5A3, COL15A1, COL18A1, COL4A1, COL4A2 |
| Genome instability | ATM, CHEK2, HUS1, MDC1, PARP1, RAD50, RAD9A, SMC1A, CCNH, CDK7, DDB1, DDB2, ERCC2, ERCC3, LIG1, PNKP, RAD23A, RAD23B, RPA1, RPA3, SLK, XAB2, XPC, APEX1, LIG3, MPG, NTHL1, POLB, UNG, XRCC1, MLH1, MSH3, MSH6, POLD3, FEN1, LIG4, PRKDC, RAD21, XRCC4, XRCC5, XRCC6, MCM2, MCM3, MCM4, MCM5, CDK1, STMN1, AIMP2, NBN, NPM1, PCNA, EGFR, KAT5, NCOA1, HDAC1, HDAC2, HDAC3, HDAC4, HDAC6, HDAC7, CBX3, NAP1L1, ATRX, CIB1, MGMT, TOP2A |
| Resisting cell death | BCL2L1, MCL1, BAX, BAK1, BAD, TRADD, FAS, CASP1, CASP2, CASP3, CASP4, CASP6, CRADD, PYCARD, NFKB1, NFKB2, NFKBIB, NFKBIE, APAF1, RIPK2, BIRC6, CARD16, DAPK1, PEA15, DAXX, FADD, ATM, CHEK2, FOXO3, SIRT1, DNMT1, HDAC1, TADA3, TP53BP2, IGF1R, NF1, RELA, CCNH, CDK1, CDK4, TSC1, MLH1, XRCC5, STAT1 |
| Sustaining proliferative signalling and evading growth suppressors | BAX, BCL2L1, CDK4, JUNB, JUND, MCL1, MET, NRAS, PIK3CA, PML, PRKCA, RAF1, SRC, STAT3, ATM, CDH1, FHIT, IGF2R, MEN1, MGMT, MLH1, NF1, NF2, S100A4, SMAD4, TSC1, XRCC1, ABL1, NFKB1, RB1, BCR, ERBB2, KRAS, SH3PXD2A |
| Inducing angiogenesis | ANG, ANGPT2, ANPEP, TYMP, JAG1, NRP1, PECAM1, PF4, SERPINF1, CXCL14, PPBP, PTN, RHOB |

**Table S1: Lists of genes associated with hallmarks of cancer.**
